# Embryonic stem cells shed new light on the developmental roles of p53

**DOI:** 10.1186/2045-3701-3-42

**Published:** 2013-10-09

**Authors:** Min Hwa Shin, Yunlong He, Jing Huang

**Affiliations:** 1 Cancer and Stem Cell Epigenetics, Laboratory of Cancer Biology and Genetics, National Cancer Institute, National Institutes of Health, 37 Convent Dr., Room 3140A, Bethesda 20814, MD, USA

**Keywords:** p53, Development, Embryonic stem cells, Cancer

## Abstract

The viability and subtle developmental defects of p53 knockout mice suggest that p53 does not play major role in development. However, contradictory evidence also exists. This discrepancy mainly results from the lack of molecular and cellular mechanisms and the general fact that p53 activation requires stresses. Recent studies of p53 in mouse and human ES cells and induced pluripotent stem (iPS) cells shed new light on the mechanisms of the developmental roles of p53. This review summarizes these new studies that support the developmental roles of p53, highlights the possible underlying molecular mechanisms, and discusses the potential relationship between the developmental roles and the tumor suppressive function of p53. In summary, the molecular mechanisms underlying the developmental roles of p53 are emerging, and the developmental roles and tumor suppressive function of p53 may be closely related.

## 

The history of the p53 field has made several turns. When it was first discovered as a SV40 binding protein, it was believed to be an oncoprotein because it is highly expressed in many types of tumors and the p53 cDNA from these tumors can transform normal cells together with H-Ras [1-6]. Later on, studies by several laboratories convincingly showed that wild type p53 is a tumor suppressor [7-9]. Because the mutation of the p53 gene occurs in more than half of human tumors, all of the earlier cloned p53 cDNAs from these tumor cells had mutations that either disrupted the activity of p53 or made p53 oncogenic. Soon afterwards, p53 mutants were found to have gain-of-functions, which explain some oncogenic phenotypes of mutated p53 cDNAs [10]. Therefore, a single amino acid change of p53 could switch it from a tumor suppressor to an oncogene. Similar to these studies in tumor cells, the studies of the developmental roles of p53 have also generated puzzling, sometimes contradictory, data. The absence of obvious developmental defects in p53 knockout mice strongly suggests that p53 is not required for development [11]. Other studies, however, suggest that p53, under certain conditions, is involved in the development of mice [12,13]. In Xenopus, p53 loss causes developmental defects by interacting with TGF beta signaling [14-16]. Recent studies using embryonic stem cells as a model system to study the function of p53 provided interesting insights into the developmental roles of p53. This review will highlight these new studies and re-visit the possible models of p53 in development.

## The potential roles of p53 in development

### p53 is involved in normal organogenesis

Given that p53 is important and highly expressed during development, it was surprising to observe that p53 knockout mice developed normally, while the adult mice quickly developed tumors [11]. Since then, it had been believed that p53 does not play a role during development. Subsequent studies by two independent groups revealed that p53 null mice are subject to subtle developmental defects with exencephaly being one of the major developmental defects and, to a lesser degree, craniofacial malformations [12,13]. These developmental defects are dependent on the genetic background and gender. Mice with 129/Sv background and females are more affected by p53 loss than C57BL/B6 and male mice. The exencephaly penetrance is about 23% in the females of 129/Sv strain. These results suggest a subtle role of p53 in neural development. It is unclear why p53 loss affects females more than males and why the penetrance of developmental defects is low. The possible roles of p53 in neuronal development have been thoroughly discussed in another review article [17].

Another organ that has obvious defects in p53 knockout mice is the testis [18]. The testes from p53 knockout mice contain multinucleated cells resulting from primary spermatocytes that fail to undergo meiosis [18]. However, this spermatogenesis defect is observed in adult mice, and it is unknown whether the abnormality initiates from the embryonic stage. Mice with a 129 genetic background are more susceptible to spermatogenesis defects. This observation is coincident with the fact that 129 mice with p53 knockout develop teratomas at a high frequency and are sterile. But more studies are needed to establish a firm connection between the roles of p53 in spermatogenesis and teratoma suppression. Recently, p53 has been shown to play a role in kidney development through activating the expression of Pax2, a critical transcription factor for kidney development [19] (Table 1). Intriguingly, Pax2 inhibits the activity of p53. Therefore, this mutual regulation forms a negative feedback loop during nephrogenesis [19]. Through genome-wide analysis, the kidney developmental pathways have been linked to clear cell renal cell carcinoma (ccRCC) [20]. Therefore, future studies need to address whether the p53/Pax2 axis is involved in the initiation or progression of ccRCC.

**Table 1:** Developmental and reproductive anomalies associated with p53 loss

**Anomalies**	**Stage**	**Stress**	**Background**	**Molecular**	**References**
				**mechanism**	
Implantation failure	Pre-implantation	Physiological	C57BL/6 J (more severe), 129/Sv and Mix	p53 regulates Lif	Hu, et al., Nature, 2007
Exencephaly	Embryonic	Physiological	129 (more severe), C57BL/6	Unclear	Armstrong, et al., Curr. Biol., 1995; Sah, et al., Nature Genetics, 1995
Renal hypoplasia	Embryonic	Physiological	C57BL/6	p53 regulates Pax2	Saifudeen, et al., Plos One, 2012
Polydactyly, megadactyly	Fetuses	X-ray	129/SvJ	Unclear	Norimura, et al., Nature Medicine, 1996
Tail anomaly	Fetuses	X-ray	129/SvJ	Unclear	Norimura, et al., Nature Medicine, 1996
Muscle	Adult	Physiological	Unknown	Unclear	Molchadsky, et al., Plos One, 2008
Testis	Adult	Physiological	129 (100% penetrance), C57BL/6X129 mix background is normal	Unclear	Rotter, et al., PNAS, 1993

### Embryonic lethality regulated by p53 in response to stresses

The most direct evidence showing that p53 is able to affect development is from the study of mdm2 knockout mice. Mdm2 knockout mice die between implantation and E5.5, around the stage of blastocysts from which embryonic stem cells are derived [21,22]. In addition, crossing Mdm2 knockout mice with p53 knockout mice completely rescued the lethal phenotype. Because ES cells are derived from blastocysts, these results provide strong biological evidence that p53 can be activated at the ES cell stage. During normal development, its activity is kept in check by Mdm2 and its homologue Mdm4 (also called MdmX). Interestingly, Mdm4 null mice die around 7.5-8.5 d.p.c. (days post coitum), suggesting that Mdm4 and Mdm2 play non-redundant roles during early development. The lethal phenotypes of both Mdm2 and Mdm4 knockout mice can be completely rescued by p53 null mice, demonstrating that p53 is involved in the developmental lethality of Mdm2 and Mdm4 knockout mice. Similar rescue and partial rescue phenotypes were observed in mES cells, blastocysts, or mice with knockouts of other genes, such as Brca1 [23-25], Brca2 [26], Rad51 [27], Cdc7 [28], Pict1 [29], L11 [30], Aurora kinase A [31], or Tsg101 [32]. Most of these genes are involved in DNA repair, cell cycle regulation, and nucleolar stress. These studies demonstrate that p53 can have developmental roles when the dysregulation of certain genes causes DNA damage, ribosomal stress, or other un-identified stresses.

### p53 regulates embryo implantation

p53 also can influence mouse embryo implantation by regulating the maternal Lif levels [33] (Table 1). Lif is an important maternal factor that facilitates embryo implantation. Loss of p53 in female mice greatly decreases the number of implanted embryos. This may represent one of the ways to decrease the reproductive ability of females with p53 defects. As mentioned above, p53 defects also cause abnormal spermatogenesis in mice [18]. Therefore, it is reasonable to speculate that loss of p53 and other p53 family members, p63 and p73, will eventually lead to reproductive disadvantage [34]. Indeed, the roles of p63 and p73 in reproduction have been reported [35,36]. Interestingly, the effect of p53 loss on embryo implantation is more severe in C57BL/6 J background, while the effect on spermatogenesis is more obvious in 129Sv background, suggesting that the genetic background of the host can influence the effect of p53 loss on implantation [18].

### p53 regulates teratogenesis in mice

An interesting study showed that p53 dependent apoptosis may be involved in X irradiation-induced teratogenesis [37]. In this study, X irradiation was used to treat implanted embryos at day 3.5 of gestation. 73% of p53+/+ embryos had early death, while 27% survived and displayed normal development. In contrast, only 44% of p53-/- embryos died early, 30% died late, 22% had developmental anomalies, and only 4% developed normally. These results indicate that p53-dependent apoptosis antagonizes teratogenesis by inducing embryonic death and involving an un-characterized “repairing” mechanism.

## Molecular and cellular mechanisms of the developmental roles of p53

The potential developmental roles of p53 have been extensively covered in another review article [38,39]. However, the molecular and cellular mechanisms of are not well characterized. Due to the introduction of novel technologies, such as genome-wide approaches, recent studies of p53 in ES cells shed some new light on this old topic [40,41]. In addition to its role in ES cells, p53 also has functions in other adult stem cells, such as muscle cell progenitors and osteoprogenitors [42,43].

The activity and levels of p53 in early embryos are higher than those in late embryos [44-46]. In vitro, the levels of p53 in ES cells decreases as ES cells differentiate [47]. Because ES cells are derived from blastocysts, they represent an excellent model system to study the early developmental role of p53 [48].

### p53 is kept in check by Mdm2, Mdm4 and delta40-p53

Without stresses, the high levels of p53 do not lead to the apoptosis or differentiation of ES cells because its activity is inhibited by Mdm2 and Mdm4 [49]. Removal of Mdm2 causes cell death at the blastocyst stage, indicating that p53 is poised for activation at the ES cell stage. Interestingly, deletion of Mdm4 leads to embryonic death at a later developmental stage. But it is possible that Mdm4 plays a cooperative role with Mdm2. A recent study suggests that Mdm4 has a function in ES cells [49].

Delta40-p53 is a naturally occurring splicing variant of p53, with an amino terminal deletion of 40 amino acids [50]. Delta40-p53 is highly expressed in ES cells and inhibits p53-dependent transcriptional activity. In addition, delta40-p53 appears to affect the Insulin Growth factor 1 receptor (IGF-1R) signaling, which has been shown to participate in the differentiation program of ES cells. It is currently unknown whether Delta40-p53 cooperates with Mdm2 and/or Mdm4 to suppress the activity of p53. It is likely that multiple distinct mechanisms exist in ES cells and embryos to maintain low activity of p53.

**Figure 1:**
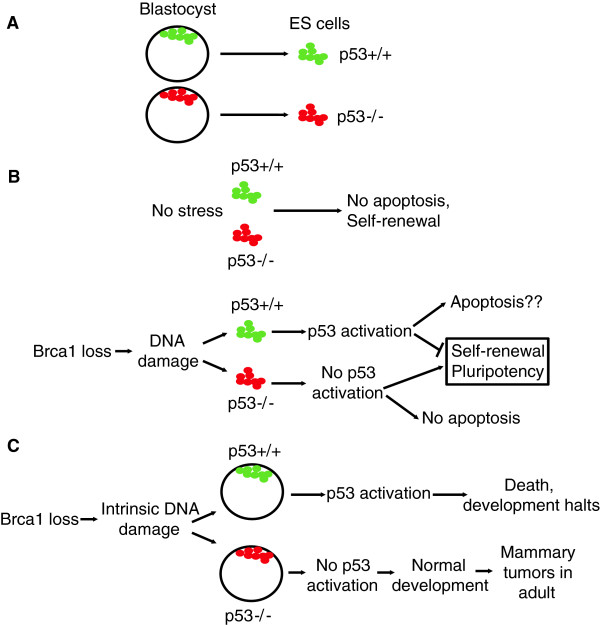
**The roles of p53 in ES cells and blastocysts. A**, Mouse ES cells (*in vitro*) are derived from blastocysts (*in vivo*) at embryonic day 3.5. **B**, In the absence of stress, p53 is inactive in ES cells. But it can be activated by exogenous or endogenous stresses, such as DNA damage, in mES cells. For example, the loss of Brca1 causes endogenous DNA damage stress, which in turn activates p53 to elicit differentiation and apoptosis of mES cells. **C**, A hypomorphic Brca1 mutant causes DNA damage in blastocysts. When p53 is intact, the DNA damage signal will activate p53 and cause the death of the blastocyst. Therefore, the developmental process is canceled. When the p53 surveillance system fails, the developmental process goes on, while the female mice develop mammary gland tumors in adulthood.

p53 can be activated in ES cell stage under certain conditions, such as Brca1 deletion. Brca1 is a protein that is involved in repairing DNA double strand breaks through homologous recombination. Its deletion in ES cells activates p53, causing the differentiation and/or apoptosis of ES cells. Therefore, ES cells containing Brca1 deletion are not viable [23,51] (Figure 1). The reduction of p53 levels in ES cells by short hairpin RNA (shRNA) can rescue the lethality caused by Brca1 loss, demonstrating that p53 activation is downstream of Brca1 loss. Similar results were observed in the Aurora kinase-p53 axis in mES cells [31].

### Functional outcomes of p53 activation in ES cells

Although it is clear that p53 induces apoptosis in human embryonic stem cells (hESCs) in response to DNA damage [52,53], it is controversial whether p53 can induce apoptosis in mES cells. Some studies showed that p53 is sequestered in the cytoplasm of mES cells, and that mES cells undergo p53-independent apoptosis in response to DNA damage [54]. Others have found that mES cells undergo p53-dependent apoptosis [55]. Different batches of sera, media, and the heterogeneity of ES cell culture could potentially explain this discrepancy.

**Figure 2:**
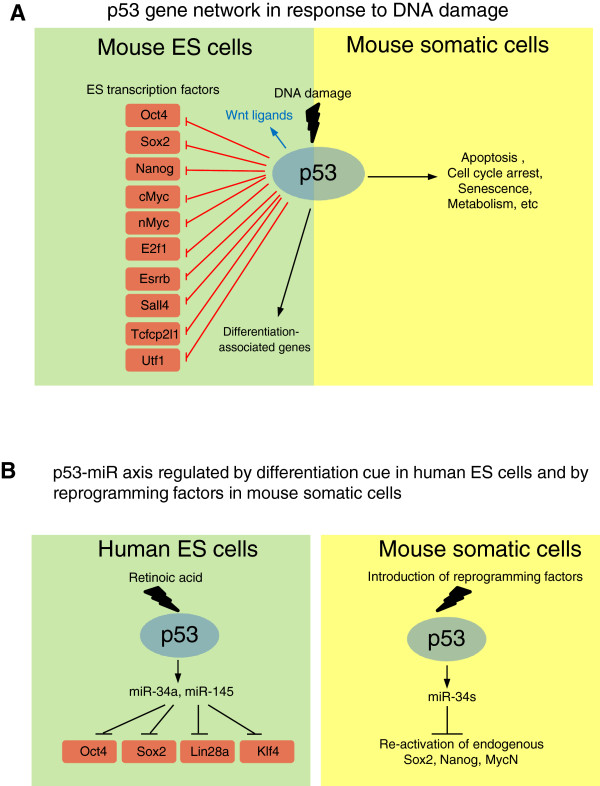
**Molecular mechanisms underlying the roles of p53 in ES cells and iPS cells. A**, In response to DNA damage, p53 can directly repress the transcription of several master regulators of mES cells. p53 also induces the transcription of several Wnt ligand genes, whose protein products are secreted from the stressed mES cells to act on neighboring cells in a paracrine manner. Interestingly, p53 up-regulates differentiation-associated genes in ES cells, suggesting that p53 signaling connects to the developmental circuitry in ES cells upon DNA damage. The cellular outcomes of p53 signaling in somatic cells are shown as a comparison. The p53-dependent cell cycle arrest and senescence are absent or have not been reported in mES cells. **B**, In human ES cells, p53-miR axis is induced by a differentiation signal, retinoic acid (RA). p53 activates miR-34a and miR-145, which in turn repress Oct4, Sox2, Lin28a, and Klf4. p53 also induces the expression of miR-34a, b, and c in mouse somatic cells upon the introduction of reprogramming factors. These miRs repress Sox2, Nanog, and nMyc, the reprogramming factors.

Despite the controversy of the apoptotic function of p53, the differentiation regulation is a well-established function of p53 in mES cells. Upon DNA damage, p53 induces the differentiation of mES cells by repressing the transcription of Nanog (Figure 2A) [56]. Apart from its pro-differentiation activity, p53 also induces the transcription of many Wnt ligand genes upon DNA damage to delay the differentiation of neighboring cells (Figure 2A) [57]. However, in differentiated cells (e.g. mouse embryonic fibroblasts) and neural progenitor cells, the induction of Wnt ligands by p53 is greatly attenuated, suggesting that the p53/Wnt axis may play a unique role in ES cells. It is possible that the p53/Wnt axis acts as a compensatory or “repairing” mechanism to restore the ES cell population [58].

It is emerging that p53 is fully functional in mES cells in response to DNA damage stress. In mES cells, p53 seems to play an important function by regulating the development-associated genes because these genes are more enriched in p53-regulated genes than apoptosis- and cell cycle-associated genes [31,40]. This observation probably represents the fact that ES cells are derived from an early developmental stage. As a well characterized cellular state, ES cells serve as a good model system for studying the early developmental events of p53 signaling. It is noteworthy that the roles of p53 in development could go beyond those discovered in ES cells, and the different epigenetic parameters at different developmental stages could affect the p53 signaling as well. Technical advances of isolating pure populations from early embryos will enable the analysis of p53 activity at different lineages in the future.

### The convergence of ES cell differentiation signaling and p53-mediated stress signaling

One of the surprising findings about the molecular mechanisms of p53 during development is that the p53 stress signaling and the developmental signaling merge at their downstream gene level [59]. Upon DNA damage, p53, like the differentiation cues during development, activates differentiation-associated genes while repressing self-renewal genes in ES cells [59]. Interestingly, in mES cells p53 is able to regulate gene transcription through binding to the enhancers far from the gene body [59]. This opens up a new avenue to study the transcriptional regulation, particularly repression, by p53. In mES cells, in response to DNA damage, p53 represses the transcription of many master regulators [59]. Surprisingly, p53 does not bind to the promoter of these master regulators. Instead, p53 binds to the distal regions to interfere with the enhancer activity. It is worth noting that the interference of enhancer is only one of the mechanisms underlying the p53-mediated repression. p53 uses many different ways to repress gene transcription in different cell types, although most of these mechanisms involve the promoter binding of p53 [60-63]. In human cells, p53 has also been shown to bind to the enhancers of development- and environment-associated genes [64]. However, it is unknown whether the binding of p53 to the enhancers in these human cells influences the transcription. The precise mechanism of how p53 interferes with the enhancer activity is unclear. A recent study indicates that p53 activates enhancer RNAs (eRNAs) to up-regulate transcription [65]. Therefore, it is reasonable to speculate that p53 could interfere with the expression of eRNA to repress transcription. Because enhancers normally drive the cell type-specific gene transcription, it is possible that p53 may repress the transcription of cell type specific genes [66]. In summary, p53 is connected to the developmental gene network and is able to regulate the network during developmental stresses, suggesting that p53 may act as a surveillance system to ensure normal development.

### p53 regulates miRNAs in human ES cells and during reprogramming of somatic cells

In human ES (hES) cells, p53 plays important roles in regulating their differentiation and apoptosis (Figure 2B) [53,67]. p53 can elicit cell cycle arrest in hES cells but not in mES cells [54,59,68,69]. The difference may result from the fact that hES cells are developmentally similar to mouse epiblast cells (around 5.5 day of the embryo) and mES cells are from 3.5-d blastocysts [70,71]. p53 promotes spontaneous apoptosis and differentiation of hES cells [53]. Upon retinoic acid (RA)-induced differentiation, p53 is activated and post-translationally modified [67]. The activated p53 then induces the transcription of miR-34a and miR-145, which can target the master regulators of ES cells, such as Oct4, Sox2, Klf4, and Lin28a [67]. A similar mode of action of p53 is also observed in the reprogramming of mouse MEF cells into induced pluripotency stem (iPS) cells (Figure 2B) [72]. There is no doubt that p53-miR plays an important role in repressing the master regulators of ES cells. Notably, the repression of the master regulators by the p53-miR axis occurs 24 hours after the RA treatment, suggesting that it is a slower process than p53-mediated interference of the enhancer activity in response to DNA damage, which occurs within 8 hours [59]. Therefore, p53 may employ multiple mechanisms to ensure the complete silencing of these master regulators. Another possibility is that 53 may behave differently in human and mouse ES cells since these two cell types represent two distinct developmental stages [70,71]. Future studies need to address the relative contribution of and relationship between the p53-miR axis and interference of enhancer activity in p53-mediated repression.

## Is the developmental role of p53 associated with the tumor suppressive function of p53?

The tumor suppressor p53 elicits many cellular functions after various stresses, such as DNA damage, hypoxia, virus infection, and unfolded protein shock. However, the connection between the cellular function and the tumor suppressive function is still unclear despite more than 30 years of study. Cell cycle arrest (reversible), senescence (irreversible cell cycle arrest), and apoptosis all have been shown to participate in tumorigenesis, suggesting that they may contribute to the tumor suppressive function of p53 [73]. Cdkn1a (also called p21 or Waf1), one of the downstream targets of p53, regulates cell cycle arrest and senescence [74]. Bbc3 (also called Puma) is required for p53-dependent apoptosis [75]. However, both Cdkn1a and Bbc3 are dispensable for the tumor suppressive function of p53, and they are not frequently mutated in human cancers, suggesting that other un-identified mechanisms are also involved [74-76]. One of the possibilities is that all the p53-mediated cellular functions are playing redundant roles during tumor suppression. The disruption of each of these cellular functions individually does not phenocopy the loss of p53 [41]. Another possibility is that we have not found the right p53 downstream targets that mediate the tumor suppressive function of p53. For example, an elegant study using a mouse model harboring a transcription-dead p53 mutant, p53 (25,26,53,54), showed that the transcriptional activity of p53 is absolutely required for its tumor suppressive function [76]. Interestingly, another p53 mutant, p53(25,26), cannot induce genes involved in acute DNA damage response while still maintaining the tumor suppressive function. These results show that the acute DNA damage associated genes cannot explain the tumor suppressive function of p53. Another possibility is that other under-appreciated functions, such as the metabolic or developmental role, of p53 are related to the tumor suppression by p53 [77]. The metabolic function of p53 has attracted much attention recently because even a p53 mutant that is defective in cell cycle arrest, apoptosis, and senescence still has tumor suppressive function [77,78]. Notably, this same p53 “super” mutant still maintains its function in regulating the metabolic pathway, suggesting that the metabolic pathway might be one of the underlying pathways responsible for the tumor suppressive function of p53. Thus far, the developmental role of p53 has not been formally linked to its tumor suppressive function. Development, by classical definition, is the time between zygote and birth, while most cancers, except for certain pediatric cancers, originate from somatic tissues. But the signaling pathways utilized by development, such as the Wnt, Notch, and TGF beta signaling, could be re-wired into a cancer program. Future efforts should be put into the investigation of the normal developmental roles of p53 and compare them to those in cancer cells. Systematic analyses show that p53-regulated genes in ES cells are playing a role in breast cancer and prostate cancer, suggesting that the role of p53 in ES cells may shed light on its tumor suppressive function [79,80].

One typical example demonstrating the relationship between the developmental role and tumor suppressive function of p53 is the functional interaction between Brca1 and p53 (Figure 1C). In development, a hypomorphic Brca1 mutant (Brca1^Δ11/Δ11^) is not compatible with life. Crossing Brca1^Δ11/Δ11^ mice with p53 heterozygous or null mice completely rescue the embryonic lethality of Brca1^Δ11/Δ11^ (Figure 1C) [24]. However, most p53+/-;Brca1^Δ11/Δ11^ female mice develop mammary tumors in their adulthood (within 6-12 months). This example shows that the failure of p53 signaling during development could also lead to tumorigenesis in the adulthood. As described above, p53 loss partially rescued the dys-regulation of other genes, such as Brca2 and Rad51, suggesting that the complete loss of these genes also activates p53-independent pathways to cause embryonic lethality. Further studies are required to determine whether the same scenario exists for other genes or in human development.

## Models of p53 in development

**Figure 3:**
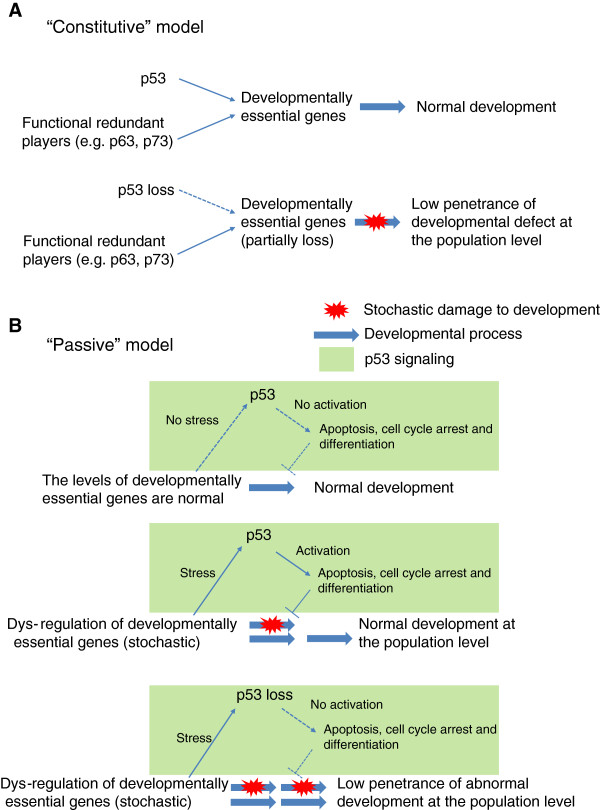
**Models of the developmental roles of p53. A**, A “constitutive” model: p53 and its functionally redundant players, presumably p63 and p73, are constitutively regulating the developmentally essential genes. The loss of p53 is partially compensated for by these redundant players. **B**, A “passive” model: p53 signaling is activated by stochastic dys-regulation of developmentally essential genes. In the basal condition, p53 is inactive but can be activated in response to developmental stresses. Therefore, p53 signaling plays a “passive” role in development. The stochastic nature of developmental stresses results in the low penetrance of developmental defects in p53 null mice.

There are at least two non-exclusive models for p53 in development (Figure 3). The first model, named the “constitutive” model, states that p53 has a constitutive or active role in regulating the developmentally essential genes (Figure 3A). Other functionally redundant players, e.g. p63 and p73, could partially compensate for the loss of p53. Therefore, the loss of p53 only causes a low penetrance of developmental defects, such as rare exencephaly. This “constitutive” model is relatively straightforward and separates the function of p53 in acute stress response from its developmental roles. This model is also supported by studies in Xenopus, in which the loss of p53 completely blocks the development [14-16]. However, it cannot explain why the complete loss of p53 also only results in low penetrance of developmental defects. The second model, called the “passive” model, combines the stress responsive function of p53 with its developmental roles (Figure 3B). In this “passive” model, p53-mediated stress responsive signaling will be activated only if the developmental process is dys-regulated. Normally, the dys-regulation of developmentally essential genes happens stochastically in embryos. It is this stochastic feature of developmental dys-regulation that causes the incomplete penetrance of developmental defects in p53 knockout mice. Although p53 activation could be detrimental to an individual embryo, it is beneficial to the whole population by ensuring that only normally developed embryos survive. We note that the two models proposed in this review are by no means comprehensive but serving as a framework for future modifications.

## Perspective

ES cells, as an excellent model for early development, have provided many novel insights into the developmental roles of p53. However, ES cells only represent a snapshot of development. p53 regulates very different sets of genes in different cell types [81]. Caution needs to be taken when extrapolating the results in ES cells into other developmental stages. The epigenetic landscapes in different cell types will shape the p53 signaling. For example, the p53-Wnt axis is tightly associated with ES cells but not in MEF and neural progenitor cells. In addition, how the p53-miR axis connects to the developmental program is largely unknown. More studies are needed to put the piece of p53 in development into the big puzzle of the tumor suppressive function of p53 in order to more effectively design therapeutic, preventive and diagnostic strategies to combat cancer. As the approaches of systems biology and genomics become increasingly available, they will provide more insights into the developmental roles of p53 and the relationship between development and cancer.

## Competing interests

The authors declare that they have no competing interests.

## Authors’ contributions

MHS, YH, and JH wrote the review. All authors read and approved the final manuscript.

## References

[B1] DeLeo AB Jay G Appella E Dubois GC Law LW Old LJ Detection of a transformation-related antigen in chemically induced sarcomas and other transformed cells of the mouse Proc Natl Acad Sci U S A 1979 76 5 2420 2424 10.1073/pnas.76.5.2420 221923 PMC383613

[B2] Crawford LV Pim DC Gurney EG Goodfellow P Taylor-Papadimitriou J Detection of a common feature in several human tumor cell lines–a 53,000-dalton protein Proc Natl Acad Sci U S A 1981 78 1 41 45 10.1073/pnas.78.1.41 6264441 PMC318985

[B3] Reich NC Levine AJ Specific interaction of the SV40 T antigen-cellular p53 protein complex with SV40 DNA Virology 1982 117 1 286 290 10.1016/0042-6822(82)90531-1 6278740

[B4] Oren M Levine AJ Molecular cloning of a cDNA specific for the murine p53 cellular tumor antigen Proc Natl Acad Sci U S A 1983 80 1 56 59 10.1073/pnas.80.1.56 6296874 PMC393308

[B5] Oren M Bienz B Givol D Rechavi G Zakut R Analysis of recombinant DNA clones specific for the murine p53 cellular tumor antigen EMBO J 1983 2 10 1633 1639 6315396 10.1002/j.1460-2075.1983.tb01637.x PMC555338

[B6] Eliyahu D Raz A Gruss P Givol D Oren M Participation of p53 cellular tumour antigen in transformation of normal embryonic cells Nature 1984 312 5995 646 649 10.1038/312646a0 6095116

[B7] Hinds P Finlay C Levine AJ Mutation is required to activate the p53 gene for cooperation with the ras oncogene and transformation J Virol 1989 63 2 739 746 2642977 10.1128/jvi.63.2.739-746.1989 PMC247745

[B8] Finlay CA Hinds PW Levine AJ The p53 proto-oncogene can act as a suppressor of transformation Cell 1989 57 7 1083 1093 10.1016/0092-8674(89)90045-7 2525423

[B9] Eliyahu D Michalovitz D Eliyahu S Pinhasi-Kimhi O Oren M Wild-type p53 can inhibit oncogene-mediated focus formation Proc Natl Acad Sci U S A 1989 86 22 8763 8767 10.1073/pnas.86.22.8763 2530586 PMC298370

[B10] Dittmer D Pati S Zambetti G Chu S Teresky AK Moore M Finlay C Levine AJ Gain of function mutations in p53 Nat Genet 1993 4 1 42 46 10.1038/ng0593-42 8099841

[B11] Donehower LA Harvey M Slagle BL McArthur MJ Montgomery CA Jr Butel JS Bradley A Mice deficient for p53 are developmentally normal but susceptible to spontaneous tumours Nature 1992 356 6366 215 221 10.1038/356215a0 1552940

[B12] Sah VP Attardi LD Mulligan GJ Williams BO Bronson RT Jacks T A subset of p53-deficient embryos exhibit exencephaly Nat Genet 1995 10 2 175 180 10.1038/ng0695-175 7663512

[B13] Armstrong JF Kaufman MH Harrison DJ Clarke AR High-frequency developmental abnormalities in p53-deficient mice Curr Biol 1995 5 8 931 936 10.1016/S0960-9822(95)00183-7 7583151

[B14] Cordenonsi M Montagner M Adorno M Zacchigna L Martello G Mamidi A Soligo S Dupont S Piccolo S Integration of TGF-beta and Ras/MAPK signaling through p53 phosphorylation Science 2007 315 5813 840 843 10.1126/science.1135961 17234915

[B15] Takebayashi-Suzuki K Funami J Tokumori D Saito A Watabe T Miyazono K Kanda A Suzuki A Interplay between the tumor suppressor p53 and TGF beta signaling shapes embryonic body axes in Xenopus Development 2003 130 17 3929 3939 10.1242/dev.00615 12874116

[B16] Cordenonsi M Dupont S Maretto S Insinga A Imbriano C Piccolo S Links between tumor suppressors: p53 is required for TGF-beta gene responses by cooperating with Smads Cell 2003 113 3 301 314 10.1016/S0092-8674(03)00308-8 12732139

[B17] Tedeschi A Di Giovanni S The non-apoptotic role of p53 in neuronal biology: enlightening the dark side of the moon EMBO Rep 2009 10 6 576 583 10.1038/embor.2009.89 19424293 PMC2711843

[B18] Rotter V Schwartz D Almon E Goldfinger N Kapon A Meshorer A Donehower LA Levine AJ Mice with reduced levels of p53 protein exhibit the testicular giant-cell degenerative syndrome Proc Natl Acad Sci U S A 1993 90 19 9075 9079 10.1073/pnas.90.19.9075 8415656 PMC47504

[B19] Saifudeen Z Liu J Dipp S Yao X Li Y McLaughlin N Aboudehen K El-Dahr SS A p53-Pax2 pathway in kidney development: implications for nephrogenesis PLoS One 2012 7 9 e44869 10.1371/journal.pone.0044869 22984579 PMC3440354

[B20] Tun HW Marlow LA Von Roemeling CA Cooper SJ Kreinest P Wu K Luxon BA Sinha M Anastasiadis PZ Copland JA Pathway signature and cellular differentiation in clear cell renal cell carcinoma PLoS One 2010 5 5 e10696 10.1371/journal.pone.0010696 20502531 PMC2872663

[B21] Montes de Oca Luna R Wagner DS Lozano G Rescue of early embryonic lethality in mdm2-deficient mice by deletion of p53 Nature 1995 378 6553 203 206 10.1038/378203a0 7477326

[B22] Jones SN Roe AE Donehower LA Bradley A Rescue of embryonic lethality in Mdm2-deficient mice by absence of p53 Nature 1995 378 6553 206 208 10.1038/378206a0 7477327

[B23] Bouwman P Aly A Escandell JM Pieterse M Bartkova J van der Gulden H Hiddingh S Thanasoula M Kulkarni A Yang Q 53BP1 loss rescues BRCA1 deficiency and is associated with triple-negative and BRCA-mutated breast cancers Nat Struct Mol Biol 2010 17 6 688 695 10.1038/nsmb.1831 20453858 PMC2912507

[B24] Xu X Qiao W Linke SP Cao L Li WM Furth PA Harris CC Deng CX Genetic interactions between tumor suppressors Brca1 and p53 in apoptosis, cell cycle and tumorigenesis Nat Genet 2001 28 3 266 271 10.1038/90108 11431698

[B25] Hakem R de la Pompa JL Elia A Potter J Mak TW Partial rescue of Brca1 (5-6) early embryonic lethality by p53 or p21 null mutation Nat Genet 1997 16 3 298 302 10.1038/ng0797-298 9207798

[B26] Ludwig T Chapman DL Papaioannou VE Efstratiadis A Targeted mutations of breast cancer susceptibility gene homologs in mice: lethal phenotypes of Brca1, Brca2, Brca1/Brca2, Brca1/p53, and Brca2/p53 nullizygous embryos Genes Dev 1997 11 10 1226 1241 10.1101/gad.11.10.1226 9171368

[B27] Lim DS Hasty P A mutation in mouse rad51 results in an early embryonic lethal that is suppressed by a mutation in p53 Mol Cell Biol 1996 16 12 7133 7143 8943369 10.1128/mcb.16.12.7133 PMC231717

[B28] Kim JM Nakao K Nakamura K Saito I Katsuki M Arai K Masai H Inactivation of Cdc7 kinase in mouse ES cells results in S-phase arrest and p53-dependent cell death EMBO J 2002 21 9 2168 2179 10.1093/emboj/21.9.2168 11980714 PMC125997

[B29] Sasaki M Kawahara K Nishio M Mimori K Kogo R Hamada K Itoh B Wang J Komatsu Y Yang YR Regulation of the MDM2-P53 pathway and tumor growth by PICT1 via nucleolar RPL11 Nat Med 2011 17 8 944 951 10.1038/nm.2392 21804542 PMC4578312

[B30] Morgado-Palacin L Llanos S Serrano M Ribosomal stress induces L11- and p53-dependent apoptosis in mouse pluripotent stem cells Cell Cycle 2012 11 3 503 510 10.4161/cc.11.3.19002 22262176

[B31] Lee DF Su J Ang YS Carvajal-Vergara X Mulero-Navarro S Pereira CF Gingold J Wang HL Zhao R Sevilla A Regulation of Embryonic and Induced Pluripotency by Aurora Kinase-p53 Signaling Cell Stem Cell 2012 11 2 179 194 10.1016/j.stem.2012.05.020 22862944 PMC3413175

[B32] Ruland J Sirard C Elia A MacPherson D Wakeham A Li L de la Pompa JL Cohen SN Mak TW p53 accumulation, defective cell proliferation, and early embryonic lethality in mice lacking tsg101 Proc Natl Acad Sci U S A 2001 98 4 1859 1864 10.1073/pnas.98.4.1859 11172041 PMC29347

[B33] Hu W Feng Z Teresky AK Levine AJ p53 regulates maternal reproduction through LIF Nature 2007 450 7170 721 724 10.1038/nature05993 18046411

[B34] Levine AJ Tomasini R McKeon FD Mak TW Melino G The p53 family: guardians of maternal reproduction Nat Rev Mol Cell Biol 2011 12 4 259 265 10.1038/nrm3086 21427767

[B35] Suh EK Yang A Kettenbach A Bamberger C Michaelis AH Zhu Z Elvin JA Bronson RT Crum CP McKeon F p63 protects the female germ line during meiotic arrest Nature 2006 444 7119 624 628 10.1038/nature05337 17122775

[B36] Tomasini R Tsuchihara K Wilhelm M Fujitani M Rufini A Cheung CC Khan F Itie-Youten A Wakeham A Tsao MS TAp73 knockout shows genomic instability with infertility and tumor suppressor functions Genes Dev 2008 22 19 2677 2691 10.1101/gad.1695308 18805989 PMC2559903

[B37] Norimura T Nomoto S Katsuki M Gondo Y Kondo S p53-dependent apoptosis suppresses radiation-induced teratogenesis Nat Med 1996 2 5 577 580 10.1038/nm0596-577 8616719

[B38] Choi J Donehower LA p53 in embryonic development: maintaining a fine balance Cell Mol Life Sci 1999 55 1 38 47 10.1007/s000180050268 10065150 PMC11146796

[B39] Hall PA Lane DP Tumor suppressors: a developing role for p53? Curr Biol 1997 7 3 R144 R147 10.1016/S0960-9822(97)70074-5 9162475

[B40] Zhang X Huang J Integrative genome-wide approaches in embryonic stem cell research Integr Biol (Camb) 2010 2 10 510 516 10.1039/c0ib00068j 20852801 PMC3400334

[B41] Li M He Y Feng X Huang J Genome-wide studies of the transcriptional regulation by p53 Biochim Biophys Acta 2012 1819 7 684 687 10.1016/j.bbagrm.2012.02.002 22348878 PMC3366165

[B42] Molchadsky A Shats I Goldfinger N Pevsner-Fischer M Olson M Rinon A Tzahor E Lozano G Zipori D Sarig R p53 plays a role in mesenchymal differentiation programs, in a cell fate dependent manner PLoS One 2008 3 11 e3707 10.1371/journal.pone.0003707 19002260 PMC2577894

[B43] Lengner CJ Steinman HA Gagnon J Smith TW Henderson JE Kream BE Stein GS Lian JB Jones SN Osteoblast differentiation and skeletal development are regulated by Mdm2-p53 signaling J Cell Biol 2006 172 6 909 921 10.1083/jcb.200508130 16533949 PMC2063734

[B44] MacCallum DE Hupp TR Midgley CA Stuart D Campbell SJ Harper A Walsh FS Wright EG Balmain A Lane DP The p53 response to ionising radiation in adult and developing murine tissues Oncogene 1996 13 12 2575 2587 9000131

[B45] Gottlieb E Haffner R King A Asher G Gruss P Lonai P Oren M Transgenic mouse model for studying the transcriptional activity of the p53 protein: age- and tissue-dependent changes in radiation-induced activation during embryogenesis EMBO J 1997 16 6 1381 1390 10.1093/emboj/16.6.1381 9135153 PMC1169735

[B46] Komarova EA Chernov MV Franks R Wang K Armin G Zelnick CR Chin DM Bacus SS Stark GR Gudkov AV Transgenic mice with p53-responsive lacZ: p53 activity varies dramatically during normal development and determines radiation and drug sensitivity in vivo EMBO J 1997 16 6 1391 1400 10.1093/emboj/16.6.1391 9135154 PMC1169736

[B47] Tam WL Lim CY Han J Zhang J Ang YS Ng HH Yang H Lim B T-cell factor 3 regulates embryonic stem cell pluripotency and self-renewal by the transcriptional control of multiple lineage pathways Stem Cells 2008 26 8 2019 2031 10.1634/stemcells.2007-1115 18467660 PMC2692055

[B48] Cao Y Regulation of germ layer formation by pluripotency factors during embryogenesis Cell & bioscience 2013 3 1 15 10.1186/2045-3701-3-15 23497659 PMC3602094

[B49] Menendez S Goh AM Camus S Ng KW Kua N Badal V Lane DP MDM4 downregulates p53 transcriptional activity and response to stress during differentiation Cell Cycle 2011 10 7 1100 1108 10.4161/cc.10.7.15090 21422812

[B50] Ungewitter E Scrable H Delta40p53 controls the switch from pluripotency to differentiation by regulating IGF signaling in ESCs Genes Dev 2010 24 21 2408 2419 10.1101/gad.1987810 21041409 PMC2964751

[B51] Chang S Biswas K Martin BK Stauffer S Sharan SK Expression of human BRCA1 variants in mouse ES cells allows functional analysis of BRCA1 mutations J Clin Invest 2009 119 10 3160 3171 10.1172/JCI39836 19770520 PMC2752086

[B52] Liu JC Guan X Ryan JA Rivera AG Mock C Agarwal V Letai A Lerou PH Lahav G High mitochondrial priming sensitizes hESCs to DNA-damage-induced apoptosis Cell Stem Cell 2013 doi:10.1016/j.stem.2013.07.018 [Epub ahead of print] 10.1016/j.stem.2013.07.018 PMC4109647 23954752

[B53] Qin H Yu T Qing T Liu Y Zhao Y Cai J Li J Song Z Qu X Zhou P Regulation of apoptosis and differentiation by p53 in human embryonic stem cells J Biol Chem 2007 282 8 5842 5852 17179143 10.1074/jbc.M610464200

[B54] Aladjem MI Spike BT Rodewald LW Hope TJ Klemm M Jaenisch R Wahl GM ES cells do not activate p53-dependent stress responses and undergo p53-independent apoptosis in response to DNA damage Curr Biol 1998 8 3 145 155 10.1016/S0960-9822(98)70061-2 9443911

[B55] De Vries A Flores ER Miranda B Hsieh HM Van Oostrom CT Sage J Jacks T Targeted point mutations of p53 lead to dominant-negative inhibition of wild-type p53 function Proc Natl Acad Sci U S A 2002 99 5 2948 2953 10.1073/pnas.052713099 11867759 PMC122453

[B56] Lin T Chao C Saito S Mazur SJ Murphy ME Appella E Xu Y p53 induces differentiation of mouse embryonic stem cells by suppressing Nanog expression Nat Cell Biol 2005 7 2 165 171 10.1038/ncb1211 15619621

[B57] Lee KH Li M Michalowski AM Zhang X Liao H Chen L Xu Y Wu X Huang J A genomewide study identifies the Wnt signaling pathway as a major target of p53 in murine embryonic stem cells Proc Natl Acad Sci U S A 2010 107 1 69 74 10.1073/pnas.0909734107 20018659 PMC2806696

[B58] Li M Huang J A new puzzling role of p53 in mouse embryonic stem cells Cell Cycle 2010 9 9 1669 1670 10.4161/cc.9.9.11596 20404507 PMC3273866

[B59] Li M He Y Dubois W Wu X Shi J Huang J Distinct regulatory mechanisms and functions for p53-activated and p53-repressed DNA damage response genes in embryonic stem cells Mol Cell 2012 46 1 30 42 10.1016/j.molcel.2012.01.020 22387025 PMC3327774

[B60] Tsai WW Nguyen TT Shi Y Barton MC p53-targeted LSD1 functions in repression of chromatin structure and transcription in vivo Mol Cell Biol 2008 28 17 5139 5146 10.1128/MCB.00287-08 18573881 PMC2519740

[B61] Nguyen TT Cho K Stratton SA Barton MC Transcription factor interactions and chromatin modifications associated with p53-mediated, developmental repression of the alpha-fetoprotein gene Mol Cell Biol 2005 25 6 2147 2157 10.1128/MCB.25.6.2147-2157.2005 15743813 PMC1061614

[B62] Murphy M Ahn J Walker KK Hoffman WH Evans RM Levine AJ George DL Transcriptional repression by wild-type p53 utilizes histone deacetylases, mediated by interaction with mSin3a Genes Dev 1999 13 19 2490 2501 10.1101/gad.13.19.2490 10521394 PMC317076

[B63] Laptenko O Prives C Transcriptional regulation by p53: one protein, many possibilities Cell Death Differ 2006 13 6 951 961 10.1038/sj.cdd.4401916 16575405

[B64] Zhu J Adli M Zou JY Verstappen G Coyne M Zhang X Durham T Miri M Deshpande V De Jager PL Genome-wide chromatin state transitions associated with developmental and environmental cues Cell 2013 152 3 642 654 10.1016/j.cell.2012.12.033 23333102 PMC3563935

[B65] Melo CA Drost J Wijchers PJ van de Werken H De Wit E Oude Vrielink JA Elkon R Melo SA Leveille N Kalluri R eRNAs are required for p53-dependent enhancer activity and gene transcription Mol Cell 2013 49 3 524 535 10.1016/j.molcel.2012.11.021 23273978

[B66] Heintzman ND Hon GC Hawkins RD Kheradpour P Stark A Harp LF Ye Z Lee LK Stuart RK Ching CW Histone modifications at human enhancers reflect global cell-type-specific gene expression Nature 2009 459 7243 108 112 10.1038/nature07829 19295514 PMC2910248

[B67] Jain AK Allton K Iacovino M Mahen E Milczarek RJ Zwaka TP Kyba M Barton MC p53 regulates cell cycle and microRNAs to promote differentiation of human embryonic stem cells PLoS Biol 2012 10 2 e1001268 10.1371/journal.pbio.1001268 22389628 PMC3289600

[B68] Song H Chung SK Xu Y Modeling disease in human ESCs using an efficient BAC-based homologous recombination system Cell Stem Cell 2010 6 1 80 89 10.1016/j.stem.2009.11.016 20074536

[B69] Zhao T Xu Y p53 and stem cells: new developments and new concerns Trends Cell Biol 2010 20 3 170 175 10.1016/j.tcb.2009.12.004 20061153

[B70] Tesar PJ Chenoweth JG Brook FA Davies TJ Evans EP Mack DL Gardner RL McKay RD New cell lines from mouse epiblast share defining features with human embryonic stem cells Nature 2007 448 7150 196 199 10.1038/nature05972 17597760

[B71] Brons IG Smithers LE Trotter MW Rugg-Gunn P Sun B Chuva de Sousa Lopes SM Howlett SK Clarkson A Pedersen RA Ahrlund-Richter L Derivation of pluripotent epiblast stem cells from mammalian embryos Nature 2007 448 7150 191 195 10.1038/nature05950 17597762

[B72] Choi YJ Lin CP Ho JJ He X Okada N Bu P Zhong Y Kim SY Bennett MJ Chen C miR-34 miRNAs provide a barrier for somatic cell reprogramming Nat Cell Biol 2011 13 11 1353 1360 10.1038/ncb2366 22020437 PMC3541684

[B73] Vousden KH Prives C Blinded by the Light: The Growing Complexity of p53 Cell 2009 137 3 413 431 10.1016/j.cell.2009.04.037 19410540

[B74] Deng C Zhang P Harper JW Elledge SJ Leder P Mice lacking p21CIP1/WAF1 undergo normal development, but are defective in G1 checkpoint control Cell 1995 82 4 675 684 10.1016/0092-8674(95)90039-X 7664346

[B75] Nakano K Vousden KH PUMA, a Novel Proapoptotic Gene, Is Induced by p53 Mol Cell 2001 7 3 683 10.1016/S1097-2765(01)00214-3 11463392

[B76] Brady CA Jiang D Mello SS Johnson TM Jarvis LA Kozak MM Kenzelmann Broz D Basak S Park EJ McLaughlin ME Distinct p53 transcriptional programs dictate acute DNA-damage responses and tumor suppression Cell 2011 145 4 571 583 10.1016/j.cell.2011.03.035 21565614 PMC3259909

[B77] Li T Kon N Jiang L Tan M Ludwig T Zhao Y Baer R Gu W Tumor suppression in the absence of p53-mediated cell-cycle arrest, apoptosis, and senescence Cell 2012 149 6 1269 1283 10.1016/j.cell.2012.04.026 22682249 PMC3688046

[B78] Liang Y Liu J Feng Z The regulation of cellular metabolism by tumor suppressor p53 Cell & bioscience 2013 3 1 9 10.1186/2045-3701-3-9 23388203 PMC3573943

[B79] Mizuno H Spike BT Wahl GM Levine AJ Inactivation of p53 in breast cancers correlates with stem cell transcriptional signatures Proc Natl Acad Sci U S A 2010 107 52 22745 22750 10.1073/pnas.1017001108 21149740 PMC3012457

[B80] Markert EK Mizuno H Vazquez A Levine AJ Molecular classification of prostate cancer using curated expression signatures Proc Natl Acad Sci U S A 2011 108 52 21276 21281 10.1073/pnas.1117029108 22123976 PMC3248553

[B81] Zhang X He Y Lee KH Dubois W Li Z Wu X Kovalchuk A Zhang W Huang J Rap2b, a novel p53 target, regulates p53-mediated pro-survival function Cell Cycle 2013 12 8 1279 1291 10.4161/cc.24364 23535297 PMC3674092

